# MAKO Robotic-Arm-Assisted Versus Conventional Dual-Incision Total Hip Arthroplasty: A Propensity-Score-Matched Retrospective Study

**DOI:** 10.3390/jcm15020405

**Published:** 2026-01-06

**Authors:** Le Wan, Chan-Young Lee, Kyung-Soon Park

**Affiliations:** Department of Orthopedic Surgery, Chonnam National University Medical School and Hwasun Hospital, Hwasun-gun 58128, Jeollanam-do, Republic of Korea; wle202302@gmail.com (L.W.); cnuhoslee@gmail.com (C.-Y.L.)

**Keywords:** total hip arthroplasty, robotic surgical procedures, propensity score matching, minimally invasive surgical procedures, treatment outcome

## Abstract

**Background:** This propensity-score-matched retrospective study compared radiographic accuracy and short-term functional outcomes between MAKO robotic-arm-assisted and conventional dual-incision minimally invasive total hip arthroplasty (THA). It was hypothesized that robotic assistance would provide superior radiographic accuracy, primarily smaller absolute deviations from the planned acetabular inclination and anteversion and a higher proportion of cups within the Lewinnek safe zone, without improving early functional outcomes. **Methods:** Consecutive patients who underwent dual-incision total hip arthroplasty were retrospectively analyzed at two affiliated institutions between March 2023 and March 2025. The study included 52 robotic-arm-assisted cases. The dual-incision technique used an anterolateral incision for acetabular preparation and cup implantation and a posterolateral incision for femoral preparation and stem implantation. Propensity score matching (1:1) generated 52 balanced pairs for age, sex, body mass index (BMI), preoperative Harris Hip Score (HHS), ASA class, and diagnosis. Operative time, blood loss, radiographic accuracy (acetabular anteversion, inclination, leg-length discrepancy [LLD], femoral and combined offsets, and stem subsidence), and functional outcomes (HHS, Oxford Hip Score [OHS], Forgotten Joint Score-12 [FJS-12]) were compared. **Results**: The robotic group achieved smaller deviations from the planned anteversion (1.15° vs. 3.0°, *p* < 0.001) and inclination (1.33° vs. 4.5°, *p* < 0.001), with a higher proportion of cups within the Lewinnek safe zone (98.1% vs. 82.7%, *p* = 0.016). Significant improvements were also seen in femoral stem subsidence (*p* = 0.006) and offset restoration, although the reduction in leg-length discrepancy did not reach statistical significance. Operative time was longer (77.8 vs. 65.0 min, *p* = 0.001), while blood loss and 6-month functional scores were comparable (HHS, *p* = 0.144; OHS, *p* = 0.328). Multivariable regression confirmed that greater deviations in acetabular orientation, higher LLD, and increased subsidence were independent predictors of poorer functional outcomes. **Conclusions:** MAKO robotic-arm assistance was associated with improved radiographic accuracy and biomechanical restoration in dual-incision THA, but no direct short-term functional advantage was observed. Greater radiographic precision was independently associated with better patient-reported outcomes, suggesting that technical precision is a key factor in optimizing early postoperative outcomes, highlighting the importance of technical accuracy in total hip arthroplasty.

## 1. Introduction

Total hip arthroplasty (THA) is a highly successful surgical intervention, providing reliable pain relief and functional restoration for patients with end-stage hip disease [1]. The development of minimally invasive surgical (MIS) techniques has sought to further improve recovery by reducing soft-tissue trauma and postoperative pain.

Among MIS approaches, the dual-incision technique offers a truly intermuscular pathway that may minimize muscle damage. However, its wider adoption has been limited by technical complexity and an associated learning curve, as highlighted in prior reports, including our previous departmental study (Tumin et al., 2014) [2]. Restricted exposure increases the risk of inaccurate component positioning, which is a major determinant of implant survival and complications such as dislocation, wear, and revision [3]. Because visualization is restricted and key steps rely more heavily on indirect landmarks and constrained instrument trajectories, the dual-incision approach may be more prone to errors in component positioning.

Robotic-arm-assisted systems, such as the MAKO platform, provide CT-based three-dimensional planning and intraoperative haptic guidance to enhance precision in implant positioning. Numerous studies have confirmed improved accuracy with robotic assistance in standard approaches to THA [4,5]. However, evidence remains limited regarding its role in technically demanding MIS techniques, particularly the dual-incision approach, where the potential for error is greater [6].

Therefore, the aim of this study was to compare radiographic accuracy and short-term functional outcomes between MAKO robotic-arm-assisted and conventional dual-incision THA in a propensity-score-matched cohort. It was hypothesized that robotic assistance would provide superior radiographic accuracy without a significant difference in early functional outcomes. This study further focuses on the dual-incision minimally invasive THA approach, a technically demanding technique characterized by limited visualization. Within this context, the robotic system may act as a compensatory tool to overcome these technical challenges, offering insights not previously addressed in studies using standard single-incision approaches.

## 2. Materials and Methods

The purpose of the study was to compare the radiographic accuracy and short-term functional outcomes between MAKO robotic-arm-assisted (Stryker, Mahwah, NJ, USA) and conventional dual-incision minimally invasive THA. The retrospective cohort comprised consecutive patients treated at two affiliated tertiary institutions between March 2023 and March 2025. This study was approved by the Institutional Review Board of Chonnam National University Hwasun Hospital (IRB No. CNUHH-2025-178, 23 September 2025); the requirement for informed consent was waived because of the retrospective design. The study adhered to the principles of the Declaration of Helsinki. As an exploratory retrospective cohort, prospective registration was not performed. No a priori sample size calculation was undertaken; all eligible consecutive patients during the study period were included to maximize statistical power.

### 2.1. Patient Selection and Matching

**Figure 1:**
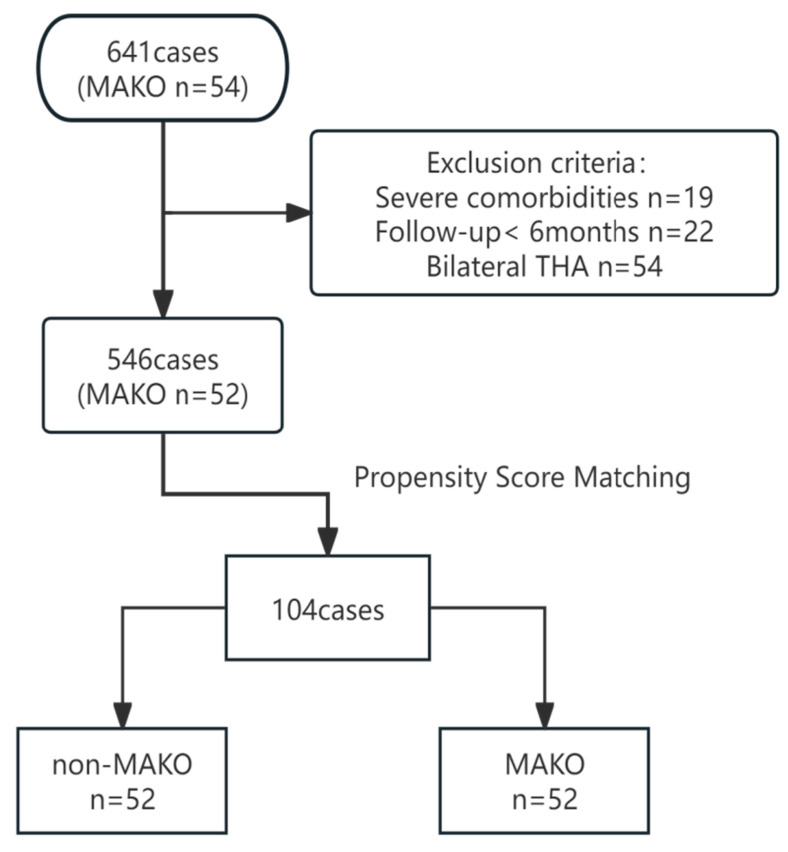
Flow diagram illustrating patient selection and propensity score matching. From 641 initial patients, exclusions were made for severe comorbidities, bilateral THA, and follow-up shorter than six months, resulting in 52 matched pairs (104 patients).

A total of 641 consecutive patients undergoing dual-incision THA during the study period were screened (Figure 1). Patients with severe comorbidities (n = 19), follow-up shorter than six months (n = 22), or simultaneous bilateral procedures (n = 54) were excluded. This resulted in 546 eligible patients, of whom 52 received MAKO-THA and 494 received dual-incision minimally invasive THA. Routine follow-up visits were scheduled at 4 weeks, 3 months, 6 months, and 12 months postoperatively, and annually thereafter when feasible.

**Figure 2:**
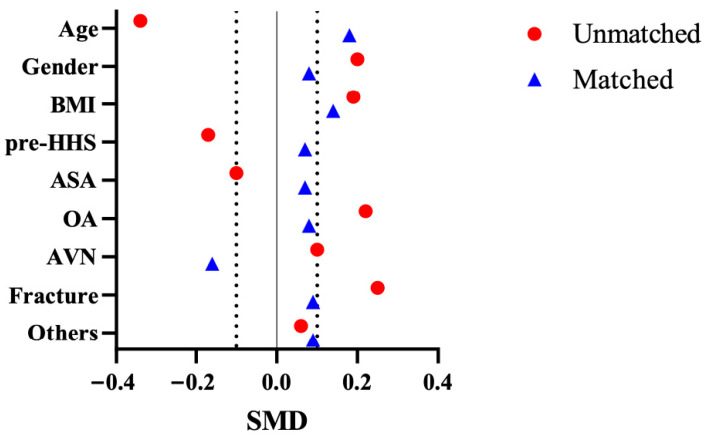
Propensity score matching (PSM) balance plot. The figure shows standardized mean differences (SMD) of baseline covariates before and after propensity score matching. Red circles represent the unmatched cohort and blue triangles the matched cohort (n = 52 pairs). The vertical line at SMD = 0 indicates perfect balance; dashed lines indicate SMD < 0.2, the commonly accepted threshold for adequate balance. After matching, all covariates showed SMDs < 0.2.

To mitigate selection bias, a 1:1 propensity score matching (PSM) was performed using age, sex, body mass index (BMI), preoperative Harris Hip Score (pre-HHS), American Society of Anesthesiologists (ASA) grade, and diagnosis as covariates. Nearest-neighbor matching with a caliper of 0.01 generated 52 well-balanced pairs for comparison (Figure 2).

All 52 matched pairs (n = 104) had complete 6-month HHS and OHS data and were used for the primary functional outcome analyses and for the HHS/OHS regression models. FJS-12 was available only among patients with ≥12 months of follow-up and was analysed in that subgroup (n = 76; conventional n = 51, MAKO n = 25).

In this ≥12-month subset, follow-up duration differed significantly between groups (conventional: 22.29 months [IQR 18–24] vs. MAKO: 17.04 months [IQR 12–21]; *p* < 0.001). The smaller number of MAKO cases contributing to ≥12-month analyses reflected limited availability of ≥12-month follow-up within the study observation window (i.e., some MAKO cases had not yet reached the 12-month timepoint), rather than loss to follow-up. Only one patient (conventional group) was lost to follow-up between the 6-month and 12-month assessments; this patient was therefore excluded from the ≥12-month subgroup analysis.

Although two hospitals were included, both are affiliated with Chonnam National University and share the same clinical system; all THA procedures were performed by the same surgical team, and perioperative blood management (including transfusion decisions) followed the same institutional practice across sites.

Osteoporosis was not a formal exclusion criterion, and bone quality was not systematically quantified with DEXA in this retrospective cohort. Standard preoperative radiographs and clinical records were reviewed as part of routine surgical planning; patients with clearly inadequate bone stock for the intended implants/technique were not selected.

### 2.2. Surgical Technique

All operations were performed by the same senior hip surgery team operating across both participating institutions to ensure consistency in surgical philosophy and technique. The dual-incision minimally invasive approach was used in all patients. Implant selection followed routine institutional availability and surgeon preference; therefore, implant manufacturers were not identical across groups. All femoral stems were implanted without cement. Supplemental acetabular screw fixation was not routinely used, but one or two screws were occasionally added at the surgeon’s discretion in elderly patients with poor bone quality to optimize initial fixation.

Implants: In the conventional (non-MAKO) group, patients received an Accolade II femoral stem (Stryker, Mahwah, NJ, USA) paired with a Lima cup (Lima Corporate, San Daniele del Friuli, Italy) or a Bencox cup (Corentec, Seoul, Republic of Korea). In this group, a Ceramic-on-Ceramic (CoC) bearing surface was utilized.

In the MAKO group, all patients received an Accolade II femoral stem paired with a Trident II acetabular cup (Stryker, Mahwah, NJ, USA). The bearing couple consisted of a fourth-generation ceramic head and an X3^®^ highly cross-linked polyethylene liner (Ceramic-on-Polyethylene, CoP). All implants in both groups were cementless.

Dual-incision minimally invasive approach: The dual-incision technique consisted of two separate skin incisions. The first incision was placed over the anterolateral aspect of the hip and was used primarily for acetabular exposure and cup implantation. An intermuscular interval was developed between the gluteus medius and the tensor fasciae latae. The second incision was placed over the posterolateral aspect of the hip and was used for femoral preparation and stem implantation. The gluteus maximus was split in line with its fibers, and an intermuscular interval was developed to access the short external rotators and proximal femur.

MAKO robotic-arm-assisted workflow: In the MAKO group, patient-specific three-dimensional (3D) surgical planning was performed based on preoperative CT scans to define acetabular cup size, target inclination/anteversion, and planned cup position. Three pelvic tracking arrays were fixed to the ipsilateral anterior superior iliac spine (ASIS) using three percutaneous pins through a small separate stab incision. The MAKO system was run using the Express workflow (software version 4.0). Pelvic registration was completed using paired-point and surface mapping according to the manufacturer’s protocol, followed by verification of registration accuracy.

Acetabular exposure was obtained through a Watson–Jones anterolateral incision. Under robotic haptic guidance, acetabular reaming and cup impaction were performed within the planned boundaries. The acetabulum was typically prepared using a 1 mm under-reaming strategy relative to the planned cup size to enhance press-fit fixation. Supplemental acetabular screws were not used routinely; when indicated, one or two screws were placed at the surgeon’s discretion in elderly patients with poor bone quality to optimize initial fixation.

Femoral preparation and stem implantation were performed through a separate posterior incision via the intermuscular interval between the gluteus medius and the piriformis, using conventional instruments (the femoral side was not navigated by the MAKO system in this workflow). Trial reduction was performed to assess stability, leg length, and offset. Intraoperative C-arm fluoroscopy was used in both groups to assist assessment of component position, leg length, and offset during trialing and final implantation.

Postoperative rehabilitation followed the same standardized protocol at both centers and did not differ between groups.

### 2.3. Outcome Measures

#### 2.3.1. Perioperative and Complication Data

Operative time (from skin incision to closure) and estimated blood loss (EBL) were recorded. Postoperative complications—including dislocation, periprosthetic fracture, nerve palsy, infection, and aseptic loosening—were systematically documented throughout follow-up.

#### 2.3.2. Estimation of Blood Loss

Patient-specific estimated blood volume (EBV) was first calculated using the Nadler formula. Total blood loss was then calculated using the Gross formula by applying the change between the most recent hemoglobin value within 7 days preoperatively and the first postoperative measurement at 24–48 h. For patients who received transfusions, the transfused volume was added to the calculated value [7].

#### 2.3.3. Radiographic Assessment

**Figure 3:**
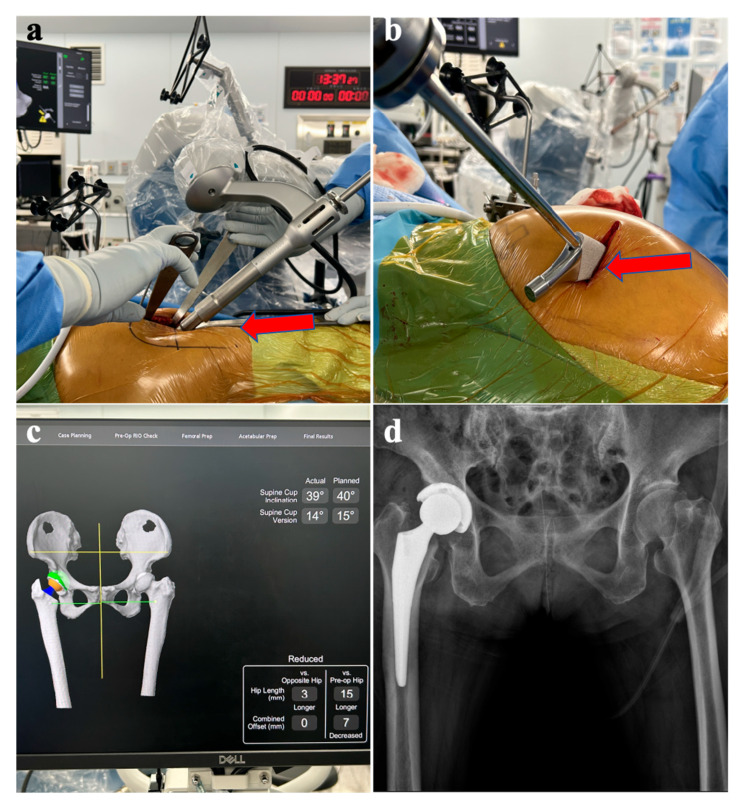
Intraoperative and postoperative images of dual-incision MAKO-assisted total hip arthroplasty. (**a**) Placement of the acetabular cup through the anterolateral incision using the MAKO robotic arm. The arrow indicates the surgical field where the acetabular component is being inserted. (**b**) Insertion of the femoral stem through the posterolateral incision. The arrow identifies the site of stem insertion. (**c**) Three-dimensional reconstruction displayed by the MAKO system after implantation of components, demonstrating the final implant position. (**d**) Postoperative anteroposterior pelvic radiograph obtained on postoperative day 2, showing satisfactory placement of the acetabular and femoral components.

Standardized standing anteroposterior (AP) pelvic radiographs were obtained postoperatively (Figure 3d) and at follow-up visits. All radiographic measurements were performed by a single experienced orthopedic surgeon using dedicated imaging software (INFINITT PACS M6; INFINITT Healthcare, Seoul, Republic of Korea) [8,9,10]. Radiographs were acquired using a standardized protocol and reviewed for acceptable pelvic positioning; images with substantial pelvic rotation/tilt that precluded reliable measurement were not used for quantitative analysis when adequate repeat imaging was unavailable.

Measured parameters included acetabular inclination (AI), acetabular anteversion (AA), leg-length discrepancy (LLD), femoral stem subsidence, and femoral and combined offsets. LLD was measured on standing AP pelvis radiographs as the inter-limb difference in perpendicular distance from the inter-teardrop line to the most prominent point of the lesser trochanter. Femoral stem subsidence (mm) was measured as the change in distance between the tip of the greater trochanter and the femoral stem shoulder from the immediate postoperative radiograph to subsequent follow-up radiographs.

The absolute difference between the planned and achieved AI and AA served as the primary measure of accuracy. Planned values were derived from preoperative planning (CT-based 3D CT plan). The percentage of acetabular cups placed within the Lewinnek safe zone (inclination 30–50°, anteversion 5–25°) was also calculated [11].

#### 2.3.4. Functional Outcomes and Clinical Significance

Functional outcomes were assessed preoperatively (HHS only) and postoperatively using the Harris Hip Score (HHS), Oxford Hip Score (OHS), and Forgotten Joint Score-12 (FJS-12). HHS and OHS were assessed at 6 months. FJS-12 was assessed at follow-up visits ≥12 months; for each patient, the last available FJS-12 score during follow-up was used for analysis.

For the OHS, the raw score (0–48) was arithmetically transformed to a 0–100 scale [12]. The Minimal Clinically Important Difference (MCID) for HHS was defined as >18 points, based on Singh et al. [13]. The Patient Acceptable Symptom State (PASS) analysis was performed for all three outcomes, with thresholds for HHS, OHS, and FJS-12 set at >89 points, >63 points, and >29 points, respectively [14,15], and was applied to the 6-month propensity-score-matched cohort analyses.

### 2.4. Data Analyses

Normality of continuous variables was assessed using the Shapiro–Wilk test. Continuous variables are presented as mean ± standard deviation (SD) when approximately normally distributed; otherwise, they are presented as median (interquartile range [IQR]). Categorical variables are presented as n (%).

In the matched cohort at 6-month follow-up, normally distributed paired data were compared using paired *t*-tests, while non-normal paired data were analyzed using the Wilcoxon signed-rank test. In the ≥12-month subgroup, the original matched-pair structure was not fully retained because ≥12-month follow-up was not available for both members of some matched pairs; therefore, comparisons were made using independent-samples *t*-tests or Mann–Whitney U tests, as appropriate. Categorical variables were analyzed with Fisher’s exact test.

Univariable linear regression was used to identify potential predictors of continuous outcomes. Variables with *p* < 0.10 in univariable analysis were entered into multivariable regression models. Details of the univariable screening and multivariable regression analyses are provided in the Supplementary Materials. Separate multivariable linear regression models were fitted for HHS, OHS, and FJS-12; the analytic cohort differed by outcome availability and follow-up timepoint as specified below. The HHS and OHS models were fitted in the 6-month propensity-score-matched cohort (52 pairs; n = 104) using complete-case analysis. The FJS-12 model was fitted in the subset of the propensity-score-matched cohort with available FJS-12 at ≥12 months (n = 76). For each patient, the last available FJS-12 score during follow-up was used, and the follow-up duration at the time of FJS-12 assessment (months) was included as a covariate.

Multicollinearity was assessed using variance inflation factors (VIFs < 10). Model fit was evaluated using adjusted R^2^ and residual diagnostics. All tests were two-tailed, with statistical significance set at *p* < 0.05. Missing data for longer-term outcomes (e.g., FJS-12) were primarily due to limited availability of ≥12-month follow-up within the study observation window; true loss to follow-up was minimal (one patient in the conventional group between the 6-month and 12-month follow-up intervals).

Primary analyses were performed in SPSS Statistics version 29.0 (IBM Corp., Armonk, NY, USA).

## 3. Results

### 3.1. Baseline Characteristics

Before propensity score matching (PSM), significant imbalances were observed between the two groups, particularly in age (mean 66.3 ± 8.3 years in the robotic group vs. 62.5 ± 13.7 years in the conventional group, *p* = 0.005).

After matching, 52 pairs (104 patients) were generated, and all baseline covariates achieved standardized mean differences (SMDs) < 0.20, indicating acceptable covariate balance. Matching balanced age, sex, and BMI as prespecified covariates in the propensity score model (Table 1, Figure 2). All matched patients (n = 104) had complete 6-month HHS and OHS data. FJS-12 was available in the ≥12-month follow-up subgroup (n = 76).

The matched cohorts were comparable in terms of age, sex, BMI, ASA classification, and diagnosis. No statistically relevant difference was observed in preoperative HHS (36.4 ± 6.2 vs. 37.0 ± 10.0, *p* = 0.677).

### 3.2. Perioperative Outcomes

The median (IQR) operative duration was 65.0 (55–75) minutes in the Non-MAKO group and 77.8 (70–85) minutes in the MAKO group (*p* = 0.001; Table 2).

Estimated blood loss was 368.1 (194–476) mL in the Non-MAKO group and 312.4 (210.7–405.4) mL in the MAKO group (*p* = 0.291; Table 2).

No intraoperative fractures, deep infections, or dislocations were observed in either group.

There were no intraoperative fractures, deep infections, or dislocations observed in either group. Regarding postoperative complications, a periprosthetic fracture occurred in one patient (1.9%) in the robotic-arm-assisted group, compared to two patients (3.8%) in the conventional group. Additionally, one case of aseptic loosening was reported in the conventional group, whereas none were observed in the robotic-arm-assisted cohort (Table 2).

### 3.3. Radiographic Outcomes at Six Months

**Figure 4:**
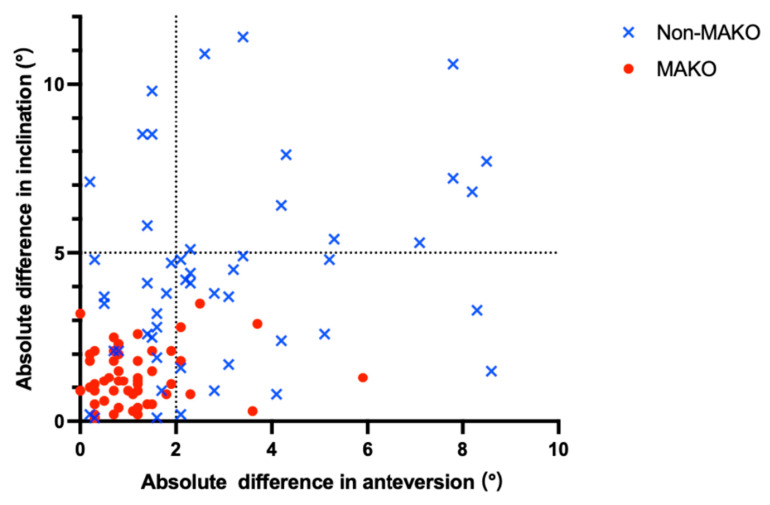
Scatter Plot of Acetabular Anteversion and Inclination Deviation. The red circles represent the MAKO group, and the blue crosses represent the non-MAKO group. The *x*-axis represents the absolute difference in acetabular anteversion (AA difference), and the *y*-axis represents the absolute difference in acetabular inclination (AI difference) for each patient. The plot demonstrates that the MAKO group (red circles) is more tightly clustered toward the origin, indicating a higher degree of accuracy in acetabular component placement compared to the non-MAKO group (blue crosses), which shows a wider distribution.

Radiographic analysis demonstrated greater accuracy of acetabular component positioning in the robotic-arm-assisted group (Table 2, Figure 4).

The absolute deviation from the preoperative plan was smaller for acetabular anteversion (median 1.15° [IQR 0.5–1.48] vs. 3.0° [1.5–4.18]; *p* < 0.001) and inclination (median 1.33° [0.65–2.0] vs. 4.5° [2.18–6.25]; *p* < 0.001).

A greater proportion of cups were positioned within the Lewinnek safe zone (98.1% vs. 82.7%; *p* = 0.016).

Accuracy of restoring hip biomechanics (radiographic restoration of leg length and femoral/combined offset relative to the preoperative plan; not range of motion) was also improved with robotic assistance.

Femoral offset difference (2.79 mm vs. 3.9 mm; *p* = 0.010) and combined offset difference (2.69 mm vs. 3.87 mm; *p* = 0.019) were smaller, and femoral stem subsidence was reduced (1.25 mm vs. 1.73 mm; *p* = 0.006).

Leg-length discrepancy was slightly smaller in the robotic group (2.66 mm vs. 3.4 mm) but did not reach statistical significance (*p* = 0.068) (Table 2).

### 3.4. Functional Outcomes at Six Months

Both groups demonstrated substantial postoperative improvement.

Median HHS was 91.9 in the robotic-arm-assisted group and 91.4 in the conventional group (*p* = 0.144).

Median OHS values were 87.9 and 87.2, respectively (*p* = 0.328).

All patients achieved the minimal clinically important difference (MCID) for HHS.

The proportion achieving the patient acceptable symptom state (PASS) was high and comparable between groups (HHS 98.1% vs. 90.4%, *p* = 0.205; OHS 100% in both groups) (Table 2).

### 3.5. Exploratory Analysis in Patients with ≥12 Months of Follow-Up

Given the unequal availability of ≥12-month follow-up between groups and the significantly different follow-up durations, this longer-term subgroup analysis should be interpreted as exploratory and hypothesis-generating. A total of 76 patients had at least one year of follow-up (25 robotic-arm-assisted, 51 conventional) (Table 3).

Follow-up duration was longer in the conventional subgroup (median 1 year 10 months [IQR 1 year 6 months to 2 years 0 months] vs. 1 year 5 months [IQR 1 year 0 months to 1 year 9 months]; *p* < 0.001).

Radiographic accuracy remained superior in the robotic-arm-assisted group, with smaller anteversion (median 1.46° vs. 3.05°; *p* < 0.001) and inclination differences (1.48° vs. 4.4°; *p* < 0.001).

Femoral stem subsidence at one year was smaller in the robotic-arm-assisted cohort (1.51 ± 0.83 mm vs. 2.32 ± 0.88 mm; *p* < 0.001).

Femoral offset difference was also lower (2.24 mm vs. 3.92 mm; *p* = 0.020), whereas combined offset difference did not reach statistical significance (*p* = 0.068).

At one year, HHS and OHS scores remained similar between groups (HHS 92.6 vs. 92.0, *p* = 0.123; OHS 88.5 vs. 88.0, *p* = 0.458).

The median FJS-12 at the latest follow-up was 75.6 in the robotic-arm-assisted group and 73.5 in the conventional group (*p* = 0.140). FJS-12 was analyzed at the latest available follow-up rather than at a fixed 12-month time point; therefore, between-group comparisons may be influenced by unequal follow-up duration. These ≥1-year subgroup findings should be interpreted cautiously given the unequal follow-up availability and significantly different follow-up duration between groups.

### 3.6. Regression Analysis

**Figure 5:**
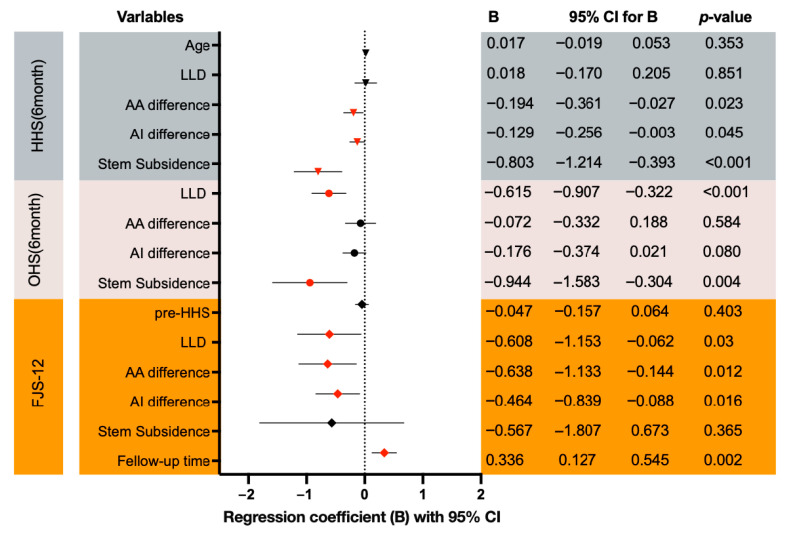
Multivariable linear regression identifying independent predictors of functional outcomes after total hip arthroplasty. Forest plots display unstandardized regression coefficients (B) with 95% confidence intervals from multivariable linear regression models for the Harris Hip Score (HHS) and Oxford Hip Score (OHS) at 6 months postoperatively and for the Forgotten Joint Score-12 (FJS-12) at the latest available follow-up. Negative coefficients indicate lower outcome scores with increasing predictor values, whereas positive coefficients indicate higher outcome scores. In the HHS model, larger deviations in acetabular anteversion and inclination and greater femoral stem subsidence were independently associated with lower 6-month HHS. In the OHS model, greater leg-length discrepancy and stem subsidence were independently associated with lower 6-month OHS. In the FJS-12 model, greater leg-length discrepancy and larger acetabular anteversion/inclination deviations were independently associated with lower FJS-12, while longer follow-up duration was independently associated with higher FJS-12.

Multivariable linear regression was conducted to identify independent predictors of postoperative functional outcomes (Figure 5).

For both the Harris Hip Score (HHS) and Oxford Hip Score (OHS), models were constructed using the six-month postoperative scores as dependent variables.

Greater acetabular anteversion deviation, larger inclination deviation, and increased femoral stem subsidence were independently associated with lower HHS values (adjusted R^2^ = 0.283).

For the OHS model, greater leg-length discrepancy and increased stem subsidence were significant predictors of worse outcomes (adjusted R^2^ = 0.378).

For the Forgotten Joint Score-12 (FJS-12), the most recent available follow-up score was analyzed with follow-up duration included as a covariate (adjusted R^2^ = 0.379).

In this model, larger acetabular orientation deviations and greater leg-length discrepancy were independently associated with lower FJS-12, whereas longer follow-up duration was associated with higher FJS-12 (B = +0.336, 95% CI 0.127–0.545; *p* = 0.002).

After adjustment for these radiographic and biomechanical parameters, robotic-arm assistance itself was not an independent predictor of short-term functional outcomes.

## 4. Discussion

The principal finding of this study is a paradox: while Mako robotic-arm assistance significantly improved radiographic precision in dual-incision minimally invasive THA, this technical superiority did not translate into measurable improvements in short-term patient-reported outcomes. This disconnect highlights the complex relationship between technical accuracy and early functional recovery and raises the broader question of how “success” in modern arthroplasty should be defined [16,17].

The lack of functional difference is likely attributable to a methodological “ceiling effect.” The profound efficacy of the dual-incision procedure resulted in 100% of patients achieving the MCID for HHS, suggesting a ceiling effect that may limit the sensitivity of traditional metrics like the HHS. As previously discussed by Wamper et al., such ceiling effects in high-performing cohorts can obscure meaningful differences between “good” and “excellent” outcomes, limiting the sensitivity of traditional metrics like the HHS [18].

Consistent with prior systematic reviews, the present findings confirm the primary technical benefit of robotic assistance in improving the accuracy of acetabular component placement [4,19,20,21]. The greater proportion of cups within the Lewinnek safe zone [11] and the reduced femoral stem subsidence suggest enhanced precision and initial stability. This technical gain, however, came at the cost of a longer operative duration, a finding consistent with studies reporting on the initial learning curve [22,23]. The additional time was primarily consumed by the setup of bone arrays and the point-to-point registration process. In our experience with the dual-incision approach, meticulous soft tissue clearance around the acetabular rim is essential to ensure that the registration probe makes direct contact with the bone, as any intervening tissue can lead to registration errors. Furthermore, the femoral tracker pins must be strategically placed to avoid interference with the skin tension of the two separate incisions or collision with the robotic arm. While the robotic system allowed for a “single-ream” technique—reaming directly with the final size—which partially offset the time spent, intraoperative fluoroscopy was still utilized as a precautionary measure during this early adoption phase to verify the final position. Although this meant that radiation reduction was not achieved in this series, the robotic navigation data consistently matched the fluoroscopic findings. In this context, the robotic system may serve as a compensatory tool to mitigate the steep learning curve associated with the dual-incision approach itself. Evidence indicates that while robotic operative time stabilizes, the accuracy of component positioning remains consistently high beyond the initial learning phase [24,25]. In our clinical experience with the dual-incision approach, several technical nuances are critical for success. First, the placement of femoral tracker pins should be strategically planned to avoid interference with the skin tension of the separate incisions and to prevent collision with the robotic arm during manipulation. Second, due to the constrained surgical window of the dual-incision technique, meticulous soft tissue clearance around the acetabular rim is essential to ensure that the registration probe makes direct contact with the bone, as any intervening tissue can lead to registration errors. We utilized a “single-ream” technique in the robotic group—reaming directly with the final target size—which partially offset the time consumed during registration. To ensure maximum safety during the early adoption phase, intraoperative fluoroscopy was still utilized in our robotic group to confirm the final prosthesis position. Although this prevented a reduction in radiation exposure in this series, the robotic navigation data consistently matched the fluoroscopic findings.

The clinical relevance of this enhanced radiographic precision is supported by the regression analyses. Greater deviation from the planned acetabular orientation and larger leg-length discrepancy were identified as independent predictors of poorer functional scores [26]. This finding provides a direct mechanistic link between the parameters optimized by the robot and the factors known to be critical for long-term implant survivorship and function, as established by previous evidence [27,28,29].

Interpretation of the Forgotten Joint Score-12 (FJS-12) warrants caution. In the ≥1-year subgroup, follow-up duration was significantly longer in the conventional group [30,31]. In our multivariable model, follow-up duration was an independent predictor of higher FJS-12, indicating that unequal follow-up timing could confound unadjusted between-group comparisons and potentially favor the group with longer follow-up. To mitigate this, we analyzed FJS-12 at the latest available follow-up and included follow-up duration as a covariate in the regression model; nevertheless, residual confounding related to differential follow-up and attrition cannot be fully excluded.

This study’s strengths include a rigorous propensity score-matching protocol and a single, experienced surgical team that minimized inter-surgeon variability. Nevertheless, limitations must be acknowledged. First, the retrospective design introduces inherent risks of selection bias. Second, because propensity score matching yields a selected analytic subset, the matched cohort may under-represent “extreme” cases, such as patients with exceptionally high BMI or complex anatomy, which may limit generalizability to the broader population. Third, although standardized preoperative radiographs were reviewed, we did not perform systematic screening for bone quality (e.g., DEXA); thus, the potential impact of osteoporosis remains unquantified. Fourth, planned component orientation was derived from 3D CT planning, whereas achieved orientation was assessed on postoperative plain radiographs; differences between imaging modalities and radiographic projection error may have influenced the calculated deviations. Fifth, we did not perform EBRA-type migration analysis; therefore, time-dependent migration of the cup or stem could not be robustly quantified beyond the predefined follow-up comparisons. Sixth, the functional scores (HHS) are self-reported and thus inherently subjective, potentially missing subtle biomechanical improvements detectable by objective gait analysis. Seventh, implant manufacturers and bearing surfaces were not identical across groups due to institutional availability and system requirements. The MAKO group utilized a Ceramic-on-Polyethylene (CoP) interface, while the conventional group received a Ceramic-on-Ceramic (CoC) system. Although CoC and CoP have different wear characteristics in the long term, previous studies have shown comparable short-term clinical and functional outcomes. All implants used in this study remained cementless and followed similar biological fixation principles. Finally, because all procedures were performed by a single experienced surgical team, the “ceiling effect” of the conventional technique may have been maximized, making technical differences less visible than they might appear in multi-center registry data.

## 5. Conclusions

MAKO robotic-arm assistance in dual-incision THA significantly improves radiographic accuracy and ensures more consistent component positioning compared to the conventional technique. While these technical advantages did not translate into superior short-term functional scores in this cohort, the enhanced precision in restoring hip biomechanics and the reduction in femoral stem subsidence represent clear immediate benefits of the robotic system. Future studies with longer follow-up are needed to determine if these early technical gains lead to improved long-term clinical outcomes.

## Figures and Tables

**Table 1:** Baseline characteristics of patients before and after propensity score matching.

	Unmatched				After Propensity Matched			
	Non-MAKO N = 494	MAKO N = 52	*p*	SMD	Non-MAKO N = 52	MAKO N = 52	*p*	SMD
Age (Yr)	62.5 ± 13.7	66.3 ± 8.3	0.005	−0.34	64.8 ± 9	66.3 ± 8.3	0.236	0.18
Gender (male/female)	228/266	19/33	0.185	0.2	21/31	19/33	0.687	0.08
BMI (kg/m^2^)	24.86 ± 4.1	24.17 ± 2.94	0.125	0.19	23.69 ± 3.81	24.17 ± 2.94	0.403	0.14
pre-HHS	35.06 ± 8.78	36.38 ± 6.17	0.165	−0.17	36.98 ± 10	36.38 ± 6.17	0.677	0.07
ASA (II/III)	431/63	47/5	0.515	−0.1	48/4	47/5	0.727	0.07
Diagnosis			0.274				0.836	
OA	202	27		0.22	25	27		0.08
AVN	205	19		0.1	23	19		−0.16
Fracture	65	3		0.25	2	3		0.09
Others	22	3		0.06	2	3		0.09

Note: *p* values are provided for descriptive purposes only and do not indicate hypothesis testing, as baseline covariates were balanced using propensity score matching.

**Table 2:** Perioperative, radiographic, and 6-month functional outcomes in the propensity score–matched cohort.

	Non-MAKO N = 52	MAKO N = 52	*p*
OP time (mins)	65.0 (55–75)	77.8 (70–85)	0.001 ^①^
EBL (mL) ^	368.1 (194–476)	312.4 (210.7–405.4)	0.291 ^②^
AA (°)	21.07 (18.33–23.4)	17.6 (15.3–19.38)	<0.001 ^②^
AI (°)	37.06 ± 4.6	39.72 ± 1.56	<0.001 ^①^
AA difference (°)	3 (1.5–4.175)	1.15 (0.5–1.48)	<0.001 ^②^
AI difference (°)	4.5 (2.18–6.25)	1.33 (0.65–2)	<0.001 ^②^
Lewinnek safe zone *	43 (82.7%)	51 (98.1%)	0.016 ^③^
Stem Subsidence (6 month) (mm)	1.73 (0.8–2.35)	1.25 (0.6–1.6)	0.006 ^②^
Femur offset difference (mm)	3.9 (2–5)	2.79 (1.25–4)	0.01 ^②^
Combined offset difference (mm)	3.87 (2–5.75)	2.69 (1–4)	0.019 ^②^
LLD (6 month) (mm)	3.4 (2.1–4.18)	2.66 (1.55–3.5)	0.068 ^②^
HHS (6 month)	91.4 (90–93)	91.88 (91–93)	0.144 ^②^
Patients achieving HHS MCID (>18), n (%)	52 (100%)	52 (100%)	1 ^③^
Patients achieving HHS PASS (>89), n (%)	47 (90.4%)	51 (98.1%)	0.205 ^③^
OHS (6 month)	87.18 (85.42–89.58)	87.86 (85.42–89.58)	0.328 ^②^
Patients achieving OHS PASS (>63), n (%)	52 (100%)	52 (100%)	1 ^③^
Combination			
Fracture	2 (3.8%)	1 (1.9%)	1 ^③^
Loosening	1 (1.9%)	0 (0%)	1 ^③^

* Defined as radiographic inclination between 30° and 50° and radiographic anteversion between 5° and 25°). ^ EBL (mL) values were calculated using the Gross formula, with patient blood volume estimated by the Nadler formula. Continuous variables are presented as mean ± SD if normally distributed, otherwise as median (IQR). Planned cup inclination/anteversion values were derived from preoperative planning (CT-based 3D plan). Deviations are reported as absolute differences between achieved postoperative radiographic measurements and the planned values. Planned targets were patient-specific and could be adjusted according to individual anatomy and stability considerations. Note: ^①^ paired *t*-test; ^②^ Wilcoxon signed-rank tests; ^③^ Fisher’s exact test.

**Table 3:** Radiographic and clinical outcomes at ≥1-year follow-up.

	Non-MAKO (N = 51)	MAKO (N = 25)	*p*
Age (Yr)	64.55 ± 8.93	64.64 ± 7.47	0.965 ^①^
Gender (male/female)	20/31 (39.2/60.8%)	10/15 (40/60%)	1 ^③^
BMI (kg/m^2^)	23.59 (20.93–25.38)	23.23 (21.51–24.09)	0.978 ^②^
BMI (kg/m^2^) (male)	22.39 (20.66–25.26)	22.92 (20.94–25.14)	0.472 ^①^
BMI (kg/m^2^) (female)	23.06 (21.5–27.73)	23.45 (22.04–23.81)	0.293 ^①^
pre-HHS	36.75 (32–42)	36.92 (33–40.5)	0.982 ^②^
Follow-up time (months)	22.29 (18–24)	17.04 (12–21)	<0.001 ^②^
ASA (II/III)	47/4	22/3	0.678 ^③^
Diagnosis			0.445 ^③^
OA	24	9	
AVN	23	11	
Fracture	2	2	
Others	2	3	
AA (°)	21.09 (18.3–23.4)	17.85 (15.65–19.35)	<0.001 ^②^
AI (°)	37.14 ± 4.61	39.51 ± 1.7	0.002 ^①^
AA difference (°)	3.05 (1.5–4.2)	1.46 (0.65–1.85)	<0.001 ^②^
AI difference (°)	4.4 (2.1–5.8)	1.48 (0.7–2.2)	<0.001 ^②^
LLD (1 yr)	3.56 (1.5–5)	2.88 (1–3.85)	0.136 ^②^
Stem Subsidence (1 yr) (mm)	2.32 ± 0.88	1.51 ± 0.83	<0.001 ^①^
Femur offset difference (mm)	3.92 (2–5)	2.24 (1–3)	0.02 ^②^
Combined offset difference (mm)	3.92 (2–5.75)	2.68 (1–4)	0.068 ^②^
HHS (1 yr)	91.98 (90–93)	92.64 (92–93)	0.123 ^②^
OHS (1 yr)	87.95 (85.42–89.58)	88.5 (86.46–89.58)	0.458 ^②^
FJS-12 (last follow-up)	73.49 (68.5–78)	75.6 (72–78.5)	0.140 ^②^

Continuous variables are presented as mean ± SD if normally distributed, otherwise as median (IQR). The test used for each variable is indicated in the Methods Section. Note: ^①^ independent-samples *t*-tests; ^②^ Mann–Whitney U tests; ^③^ Fisher’s exact test. This ≥ 1-year analysis represents an exploratory subgroup (unmatched) due to loss of the matched-pair structure with attrition.

## Data Availability

The datasets generated and analyzed during the current study are not publicly available due to patient privacy concerns but are available from the corresponding author upon reasonable request.

## Supplementary Materials

The following supporting information can be downloaded at: https://www.mdpi.com/article/10.3390/jcm15020405/s1, The Supplementary Materials include detailed univariable and multivariable regression analyses.

## Abbreviations

The following abbreviations are used in this manuscript:

THA	Total hip arthroplasty
MIS	Minimally invasive surgical
BMI	Body mass index
ASA	American Society of Anesthesiologists
LLD	Leg-length discrepancy
MCID	Minimal Clinically Important Difference
PASS	Patient Acceptable Symptom State
AP	Anteroposterior
EBV	Estimated blood volume
EBL	Estimated blood loss
IQR	Interquartile range
SMD	Standardized mean difference
VIF	Variance inflation factor
