# Supplementary material for: MAKO Robotic-Arm-Assisted Versus Conventional Dual-Incision Total Hip Arthroplasty: A Propensity-Score-Matched Retrospective Study
Source: J Clin Med. 2026 Jan 6;15(2):405. doi: 10.3390/jcm15020405 (PMC12841923; doi:10.3390/jcm15020405)

HHS (6month)

Variable	β(Standardized)	B	95%CI for B		p-value
Age	.172	.037	-.005	.078	.081
Sex(Female vs Male)	1.88013	.081	-.311	.474	0.685
BMI	-.067	-.037	-.144	.070	.496
AVN vs OA	1.88121	.044	-.345	.432	0.826
Frature vs OA	1.88082	.136	-.756	1.028	0.765
Others vs OA	1.86892	-.537	-1.431	.359	0.240
pre-HHS	-.004	-.001	-.045	.043	.971
ASA	1.87293	-.338	-1.017	.343	0.331
LLD	-.258	-.248	-.431	-.066	.008
AA difference	-.328	-.298	-.466	-.129	<.001
AI difference	-.370	-.250	-.374	-.127	<.001
Femur Offset	.036	.032	-.140	.203	.714
Combinde Offset	-.032	-.024	-.168	.120	.744
Stem Subsidence	-.440	-.923	-1.294	-.553	<.001

OHS (6month)

Variable	β(Standardized)	B	95%CI for B		p–value
Age	.129	.046	–.024	.116	.192
sex(female vs male)	1.88013	.081	–.311	.474	0.342
BMI	.045	.041	–.139	.221	.651
AVN vs OA	1.88121	.044	–.345	.432	0.826
Frature vs OA	1.88082	.136	–.756	1.028	0.765
Others vs OA	1.86892	–.537	–1.431	.359	0.240
pre–HHS	–.085	–.032	–.105	.041	.388
ASA	1.87293	–.338	–1.017	.343	0.331
LLD	–.556	–.897	–1.161	–.634	<.001
AA difference	–.193	–.294	–.587	.000	.050
AI difference	–.309	–.351	–.563	–.139	.001
Femur Offset	.099	.145	–.141	.432	.316
Combinde Offset	–.024	–.029	–.271	.213	.811
Stem Subsidence	–.499	–1.760	–2.360	–1.161	<.001

FJS 12

Variable	β(Standardized)	B	95%CI for B		p–value
Age	.057	.036	–.111	.184	.625
Sex(Female vs Male)	5.44636	–.177	–.632	.280	0.449
BMI	–.042	–.064	–.421	.293	.721
AVN vs OA	5.45909	.110	–.338	.557	0.632
Frature vs OA	5.45248	.326	–.673	1.323	0.523
Others vs OA	5.40586	–.597	–1.498	.308	.196
Follow–up time (month)	.240	.271	.018	.525	.036
pre–HHS	–.218	–.127	–.259	.004	.058
ASA	5.44886	–.280	–1.050	.491	0.477
LLD	–.429	–1.068	–1.589	–.547	<.001
AA difference	–.394	–.962	–1.481	–.442	<.001
AI difference	–.414	–.762	–1.151	–.374	<.001
Femur Offset	.075	.174	–.363	.710	.520
Combinde Offset	.066	.135	–.338	.608	.571
Stem Subsidence	–.278	–1.587	–2.859	–.315	.015

Multivariable Regression

Screening of candidate variables

All candidate variables were initially tested by univariate analysis, and those with p < 0.10 were included in the multivariate regression model

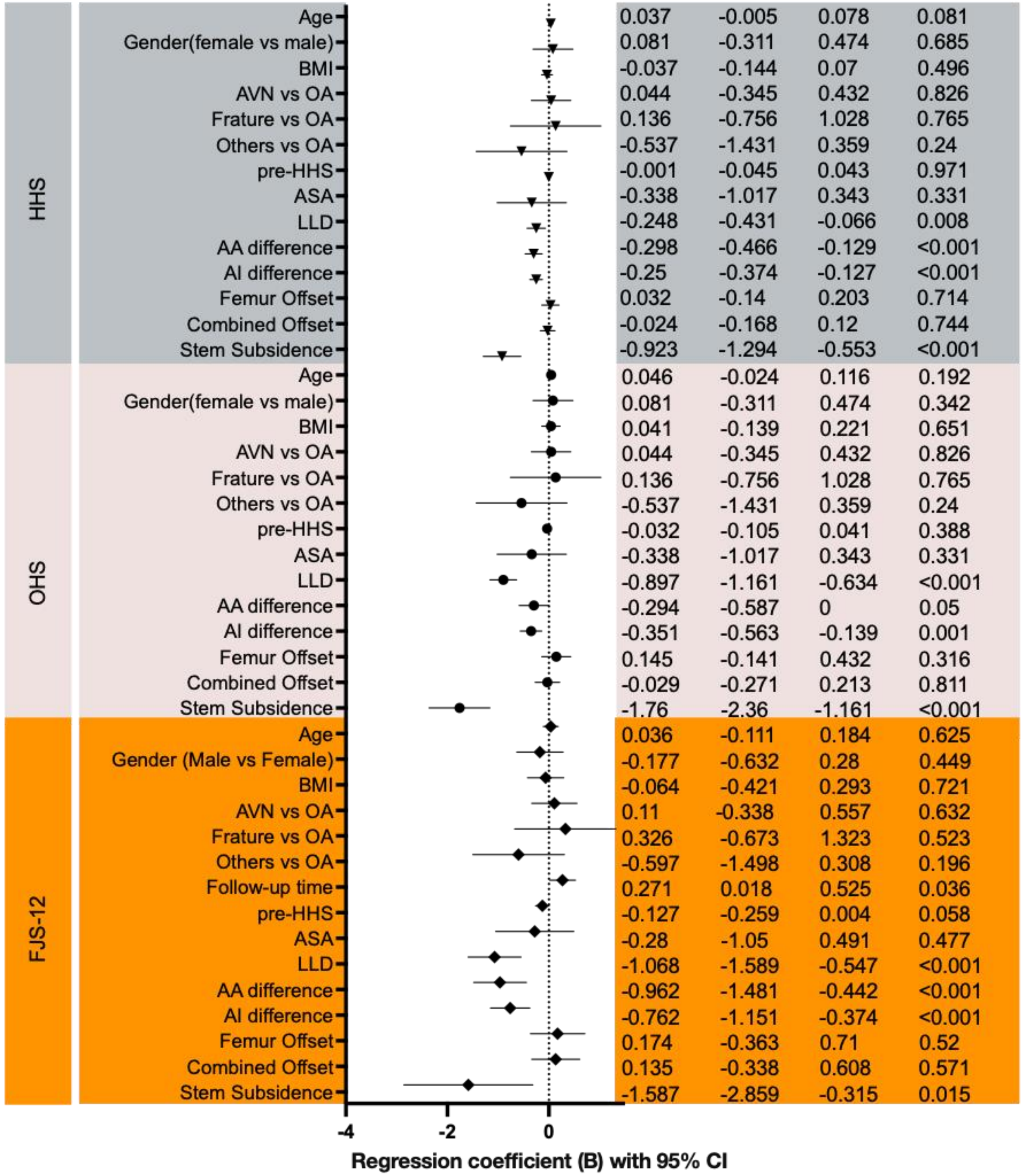

	Variables	B	95% CI for B		*p*-value
HHS	Age	0.037	-0.005	0.078	0.081
	Gender(female vs male)	0.081	-0.311	0.474	0.685
	BMI	-0.037	-0.144	0.07	0.496
	AVN vs OA	0.044	-0.345	0.432	0.826
	Frature vs OA	0.136	-0.756	1.028	0.765
	Others vs OA	-0.537	-1.431	0.359	0.24
	pre-HHS	-0.001	-0.045	0.043	0.971
	ASA	-0.338	-1.017	0.343	0.331
	LLD	-0.248	-0.431	-0.066	0.008
	AA difference	-0.298	-0.466	-0.129	<0.001
	AI difference	-0.25	-0.374	-0.127	<0.001
	Femur Offset	0.032	-0.14	0.203	0.714
	Combined Offset	-0.024	-0.168	0.12	0.744
	Stem Subsidence	-0.923	-1.294	-0.553	<0.001
OHS	Age	0.046	-0.024	0.116	0.192
	Gender(female vs male)	0.081	-0.311	0.474	0.342
	BMI	0.041	-0.139	0.221	0.651
	AVN vs OA	0.044	-0.345	0.432	0.826
	Frature vs OA	0.136	-0.756	1.028	0.765
	Others vs OA	-0.537	-1.431	0.359	0.24
	pre-HHS	-0.032	-0.105	0.041	0.388
	ASA	-0.338	-1.017	0.343	0.331
	LLD	-0.897	-1.161	-0.634	<0.001
	AA difference	-0.294	-0.587	0	0.05
	AI difference	-0.351	-0.563	-0.139	0.001
	Femur Offset	0.145	-0.141	0.432	0.316
	Combined Offset	-0.029	-0.271	0.213	0.811
	Stem Subsidence	-1.76	-2.36	-1.161	<0.001
FJS-12	Age	0.036	-0.111	0.184	0.625
	Gender (Male vs Female)	-0.177	-0.632	0.28	0.449
	BMI	-0.064	-0.421	0.293	0.721
	AVN vs OA	0.11	-0.338	0.557	0.632
	Frature vs OA	0.326	-0.673	1.323	0.523
	Others vs OA	-0.597	-1.498	0.308	0.196
	Follow-up time	0.271	0.018	0.525	0.036
	pre-HHS	-0.127	-0.259	0.004	0.058
	ASA	-0.28	-1.05	0.491	0.477
	LLD	-1.068	-1.589	-0.547	<0.001
	AA difference	-0.962	-1.481	-0.442	<0.001
	AI difference	-0.762	-1.151	-0.374	<0.001
	Femur Offset	0.174	-0.363	0.71	0.52
	Combined Offset	0.135	-0.338	0.608	0.571
	Stem Subsidence	-1.587	-2.859	-0.315	0.015

HHS
R^2=0.283

Variable	B(Unstandardized)	95%CI for B		β(Standardized)	p–value
Age	.017	–.019	.053	.079	.353
LLD	.018	–.170	.205	.018	.851
AA difference	–.194	–.361	–.027	–.214	.023
AI difference	–.129	–.256	–.003	–.191	.045
Stem subsidence	–.803	–1.214	–.393	–.382	<.001

OHS
R^2=0.378

Variable	B(Unstandardized)	95%CI for B		β(Standardized)	p–value
LLD	–.615	–.907	–.322	–.381	<.001
AA difference	–.072	–.332	.188	–.047	.584
AI difference	–.176	–.374	.021	–.155	.080
Stem Subsidence	–.944	–1.583	–.304	–.268	.004

FJS-12

R^2=0.379

Variable	B(Unstandardized)	95%CI for B		β(Standardized)	p-value
pre-HHS	-.047	-.157	.064	-.080	.403
LLD	-.608	-1.153	-.062	-.244	.030
AA difference	-.638	-1.133	-.144	-.261	.012
AI difference	-.464	-.839	-.088	-.252	.016
Stem subsidence	-.567	-1.807	.673	-.099	.365
Follow-up time	.336	.127	.545	.298	.002

Multivariable Regression

HHS: AA difference, AI difference, and femoral stem subsidence were significant independent predictors. (R^2=0.283)

OHS: LLD and femoral stem subsidence were significant independent predictors. (R^2=0.378)

FJS-12: LLD, AA difference, and AI difference were significant independent predictors. (R^2=0.379)

Fig 5

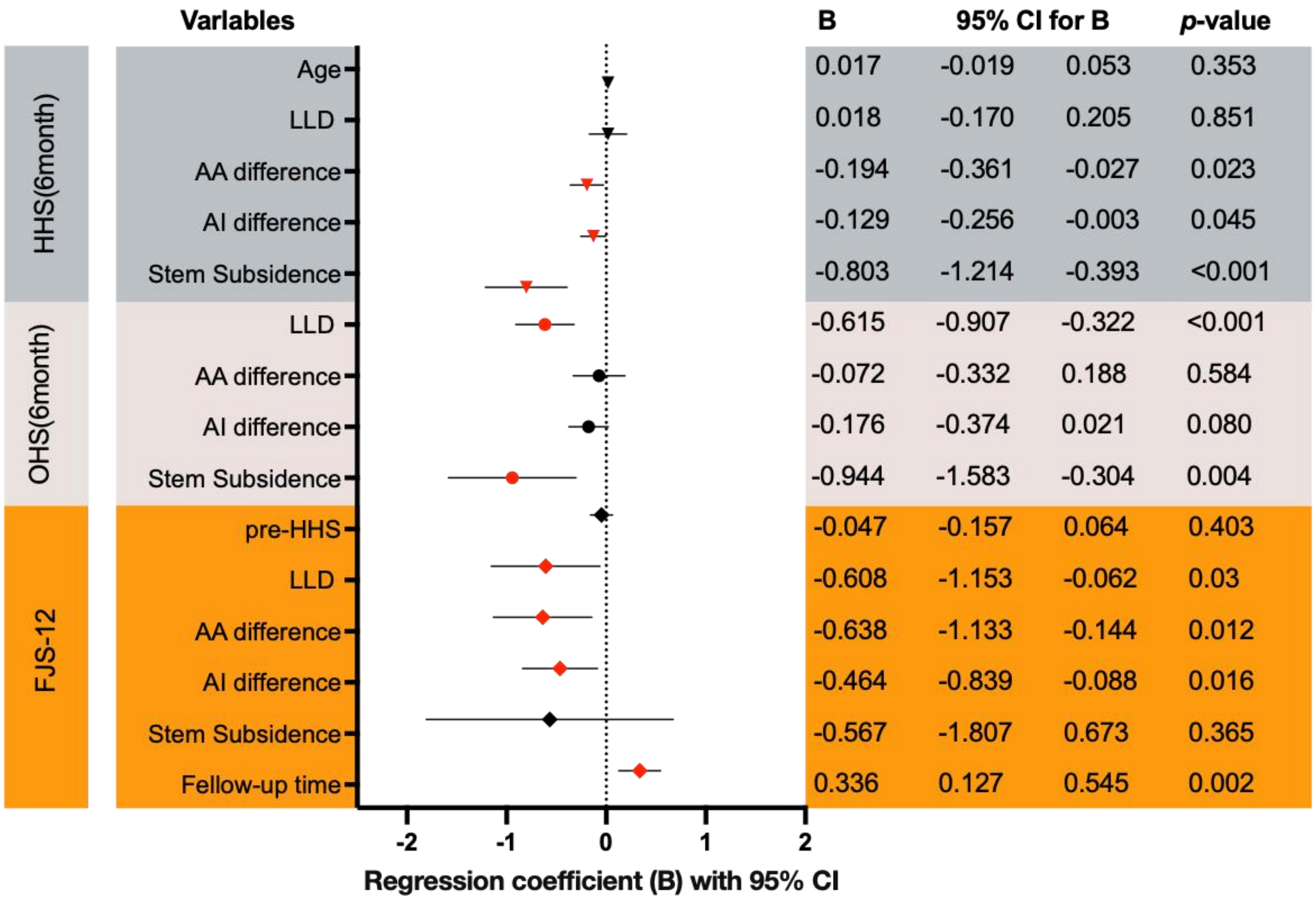

Supplement: Supplementary file 1 [file jcm-15-00405-s001.zip › jcm-4039867-supplementary.pdf]
